# Topoisomerase IIα Binding Domains of Adenomatous Polyposis Coli Influence Cell Cycle Progression and Aneuploidy

**DOI:** 10.1371/journal.pone.0009994

**Published:** 2010-04-02

**Authors:** Yang Wang, Robert J. Coffey, Neil Osheroff, Kristi L. Neufeld

**Affiliations:** 1 Department of Molecular Biosciences, University of Kansas, Lawrence, Kansas, United States of America; 2 Department of Medicine, Vanderbilt University School of Medicine, Nashville, Tennessee, United States of America; 3 Department of Cell and Developmental Biology, Vanderbilt University School of Medicine, Nashville, Tennessee, United States of America; 4 Department of Biochemistry, Vanderbilt University School of Medicine, Nashville, Tennessee, United States of America; 5 Department of Veterans Affairs Medical Center, Nashville, Tennessee, United States of America; University of Edinburgh, United Kingdom

## Abstract

**Background:**

Truncating mutations in the tumor suppressor gene *APC* (*Adenomatous Polyposis Coli*) are thought to initiate the majority of colorectal cancers. The 15- and 20-amino acid repeat regions of APC bind β-catenin and have been widely studied for their role in the negative regulation of canonical Wnt signaling. However, functions of APC in other important cellular processes, such as cell cycle control or aneuploidy, are only beginning to be studied. Our previous investigation implicated the 15-amino acid repeat region of APC (M2-APC) in the regulation of the G2/M cell cycle transition through interaction with topoisomerase IIα (topo IIα).

**Methodology/Principal Findings:**

We now demonstrate that the 20-amino acid repeat region of APC (M3-APC) also interacts with topo IIα in colonic epithelial cells. Expression of M3-APC in cells with full-length endogenous APC causes cell accumulation in G2. However, cells with a mutated topo IIα isoform and lacking topo IIβ did not arrest, suggesting that the cellular consequence of M2- or M3-APC expression depends on functional topoisomerase II. Both purified recombinant M2- and M3-APC significantly enhanced the activity of topo IIα. Of note, although M3-APC can bind β-catenin, the G2 arrest did not correlate with β-catenin expression or activity, similar to what was seen with M2-APC. More importantly, expression of either M2- or M3-APC also led to increased aneuploidy in cells with full-length endogenous APC but not in cells with truncated endogenous APC that includes the M2-APC region.

**Conclusions/Significance:**

Together, our data establish that the 20-amino acid repeat region of APC interacts with topo IIα to enhance its activity *in vitro*, and leads to G2 cell cycle accumulation and aneuploidy when expressed in cells containing full-length APC. These findings provide an additional explanation for the aneuploidy associated with many colon cancers that possess truncated APC.

## Introduction

Mutation of the tumor suppressor *Adenomatous Polyposis coli (APC)* gene is considered an initiating event in over 80% of all colorectal cancers [1]. Mutations in *APC* have also been associated with chromosomal instability and aneuploidy in early polyps from FAP (Familial Adenomatous Polyposis) patients [2], [3], [4]. Although the ability of APC to suppress canonical Wnt signaling by targeting β-catenin is critical for APC to suppress tumorigenesis [5], [6], [7], [8], [9], accumulating evidence suggests that APC likely suppresses tumor development through pathways besides inhibiting canonical Wnt signaling [see review [10]].

Among the multiple functions of APC identified is cell cycle regulation [11], [12], [13], [14], [15], [16], [17]. APC involvement in G1/S is attributed to its recognized role in canonical Wnt signaling. APC participation in the G2/M transition involves interaction with topoisomerase IIα (topo IIα) [18]. However, the mechanism by which APC regulates the G2-M cell cycle transition is poorly understood.

Besides being an enzyme that catalyzes DNA topology changes [19], [20], [21], [22], topo IIα is also a critical regulator of one G2/M checkpoint during cell division, the decatenation checkpoint [23]. Inhibition of topo IIα activity leads to initiation of the G2 decatenation checkpoint, resulting in G2 cell cycle arrest [23]. Topo IIβ, a closely related protein, has a similar amino acid sequence and activity as topo IIα, but is dispensable for cell cycle control [24], [25], [26].

Previously, we found full-length endogenous APC interacts with endogenous topo IIα but not with topo IIβ [18]. Expression of a central fragment of APC that binds topo IIα led to cell cycle accumulation in G2, independent of β-catenin [18]. Thus, we concluded that nuclear APC interacts with topo IIα and thus, might be involved in the regulation of cell cycle progression. In the current study, we identify a second domain in the central portion of APC that specifically binds to topo IIα but not topo IIβ. Both APC central domains dramatically impact the activity of topo IIα *in vitro*. Cell lines with full-length endogenous APC that express either of the APC domains capable of modifying topo IIα activity accumulate in G2 and display increased aneuploidy. Using a cell line lacking topo IIβ and with a mutant isoform of topo IIα, we demonstrate that cell cycle arrest triggered by expression of middle APC fragments requires normal expression of endogenous topo IIα. Our data implies that full-length APC can participate in the topo IIα-mediated regulation of the G2-M transition.

## Results

### Two central regions of APC bind topo IIα

**Figure 1:**
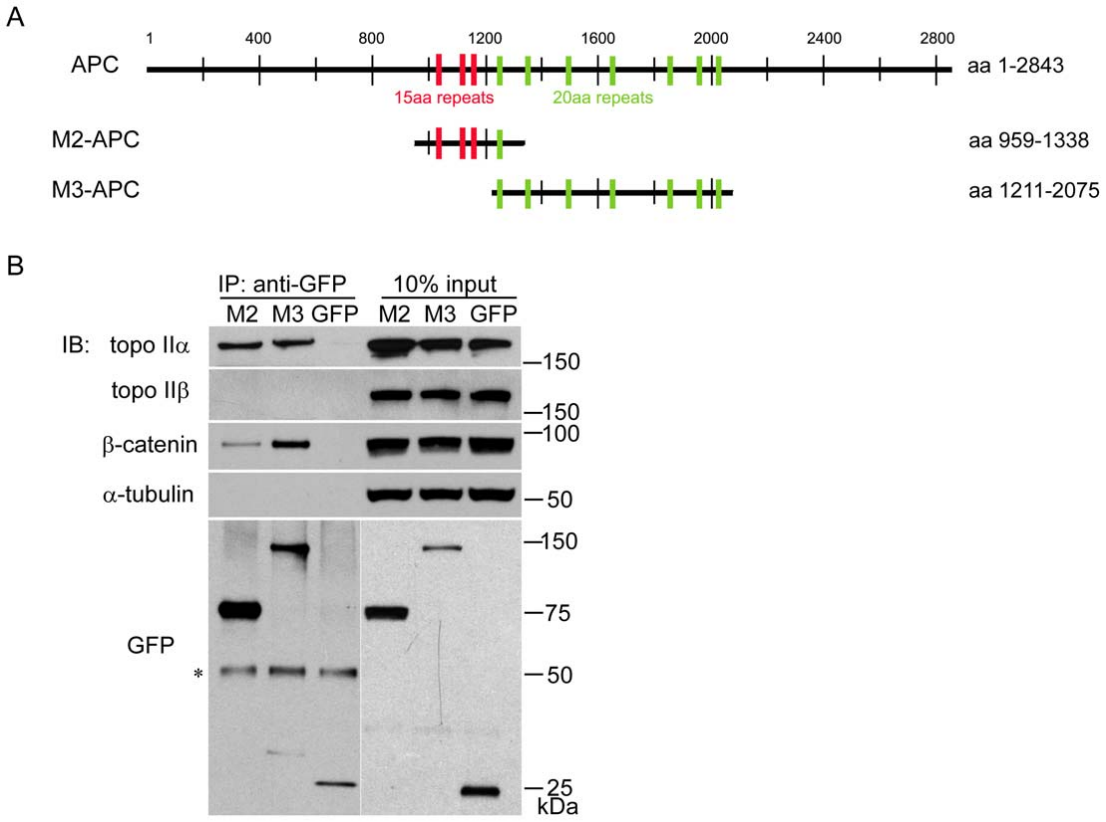
Both 15aa and 20aa repeat regions of APC interact with topo IIα. (A) Schematic diagram of APC protein showing location of M2 and M3-APC fused to the C-terminus of EGFP and used in all cell studies. (B) A GFP antibody was used to immunoprecipitate (IP) EGFP from HCT116βw cells expressing either EGFP-M2-APC, EGFP-M3-APC or EGFP. Immunoblots (IB) were probed using antibodies indicated to the left of the gel. Ten percent input equals 10 µg total protein. Topo IIα co-precipitates with both M2-APC and M3-APC, but not with EGFP. Topo IIβ does not co-precipitate with M2- or M3-APC, but β-catenin does co-precipitate with both. Blots probed for α-tubulin served as a loading control for the input samples and a negative control for the immunoprecipitations. (_*_) marks migration of antibody heavy chain. Representative blots from three independent experiments are shown.

Previously, we identified an interaction between endogenous APC and topo IIα [18]. Exogenous expression of the 15-amino acid repeat region of APC (M2-APC) that interacts with topo IIα led to G2 cell cycle arrest. In the present study, we investigated whether the 20-amino acid repeat region of APC (M3-APC) also interacts with topo IIα. Topo IIα specifically co-precipitates with both EGFP-fused M2- and M3-APC (Figure 1B, top blot). However, topo IIβ does not co-precipitate with either APC fragment under the same experimental conditions (Figure 1B, second blot). EGFP-M2-APC encompasses APC amino acids 959–1338 while EGFP-M3-APC contains amino acids 1211–2075 (Figure 1A). The M3 region contains two mono-partite nuclear localization signals [27]. Together, these two regions contain all 15- and 20-amino acid repeats of APC that mediate β-catenin binding and downregulation. Therefore, β-catenin co-precipitation with both M2- and M3-APC, served as a positive control (Figure 1B, third blot).

### A functional interaction between purified M2- or M3-APC and topo IIα

**Figure 2:**
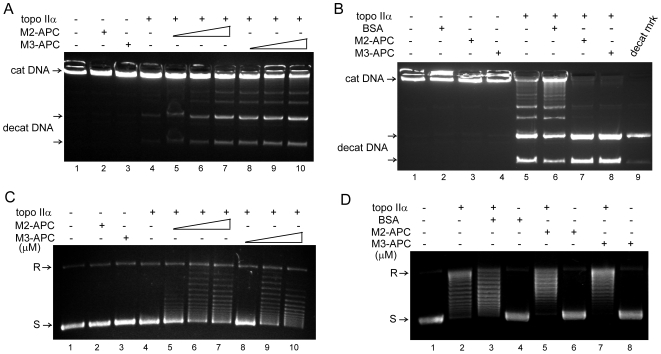
Recombinant M2- and M3-APC each enhance topo IIα activity *in vitro*. (A) Purified recombinant human topo IIα (0.12 µM) could slightly decatenate catenated DNA (catDNA) (lane 4). Addition of increasing amounts (0.12, 0.24, and 0.6 µM) of purified recombinant M2-APC (amino acid 1000–1326, lanes 5–7) or non-overlapping M3-APC (amino acid 1330–2058, lanes 8–10) resulted in progressively enhanced topo IIα DNA decatenation activity. M2-, or M3-APC (0.6 µM) alone did not display decatenation activity in the absence of topo IIα(lanes 2 and 3, respectively). (B) Using a higher concentration of topo IIα (0.18 µM) that displays slightly more activity in the absence of other proteins, the addition of M2- and M3-APC (0.18 µM) enhances topo IIα activity (lanes 7 and 8, respectively). In contrast, BSA (0.18 µM) did not enhance the DNA decatenation activity of topo IIα (lane 6). cat DNA, catenated kinetoplast DNA (kDNA); decat DNA, decatenated kDNA. (C) Purified recombinant human topo IIα (0.35 µM) could slightly relax supercoiled pBR322 plasmid DNA (lane 4). Addition of increasing amounts (0.35, 0.70, and 1.35 µM) of purified recombinant M2-APC (lanes 5–7) or M3-APC (lanes 8–10) resulted in progressively relaxed plasmids as indicated by slower migrating bands. M2-, or M3-APC (0.70 µM) did not display relaxation activity in the absence of topo IIα (lanes 2 and 3, respectively). (D) Under conditions where topo IIα displayed moderate plasmid relaxation activity, even in the absence of other proteins (lane 2), addition of BSA (0.35 µM) did not enhance this activity (lane 3), whereas addition of either M2- or M3-APC did (note reduction in faster migrating, highly supercoiled forms of DNA in lanes 5 and 7 compared to lane 2). (A–D) Representative assays from at least four independent experiments are shown.

Given that exogenous M2- and M3-APC each interact with endogenous topo IIα, we tested whether both APC fragments would also influence two different reactions catalyzed by topo IIα. Purified non-overlapping recombinant M2- and M3-APC fragments each enhanced the ability of purified topo IIα to resolve highly catenated kinetoplast DNA into decatenated mini DNA circles *in vitro* (Figure 2A and 2B). Neither purified M2- nor M3-APC showed decatenation activity in the absence of topo IIα (Figure 2A and 2B). Topo IIα enzyme can also convert supercoiled DNA into relaxed circular DNA and this relaxation activity was enhanced by addition of purified M2- or M3-APC (Figure 2C). Neither purified M2- nor M3-APC relaxed the supercoiled DNA in the absence of topo IIα (Figure 2C). While it was clear that purified M2- or M3-APC enhanced both decatenation and relaxation activities of purified topo IIα, we wanted to eliminate the possibility that these effects were due to increased total protein concentration in the reactions and were instead specific properties of M2 and M3-APC. Purified BSA did not enhance topo IIα activities (Figures 2B and 2D). In contrast, when reactions performed such that the purified topo IIα alone displayed moderate activity, the addition of purified BSA protein slightly inhibited both decatenation and relaxation activities of topo IIα (Figure 2B and 2D). These *in vitro* assays provide additional support for a functional interaction between APC and topo IIα. Furthermore, purified M2- and M3-APC had no area of overlap, and yet each interacted with purified topo IIα. We conclude that although the EGFP-M3-APC used for cell studies overlaps slightly with the EGFP-M2-APC, this area of overlap is not solely responsible for the topo IIα interaction and M2- and M3-APC can each interact with and affect topo IIα independently.

### Expression of M2- or M3-APC results in G2 cell cycle arrest

**Figure 3:**
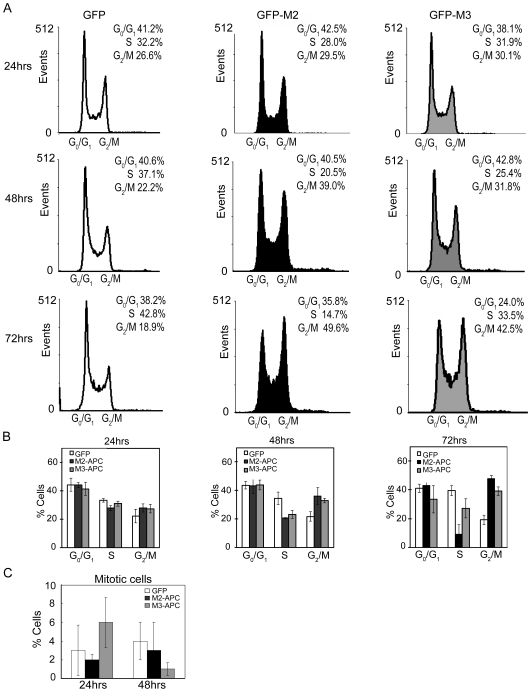
Cells expressing M2- or M3-APC progressively accumulate in G2. (A) Histograms showing representative FACS displays of cell cycle distribution assessed by Hoechst blue staining at 24, 48, and 72 hours post-transfection with expression constructs for EGFP fused M2- or M3-APC, or EGFP alone. Only EGFP-positive cells are displayed. (B) Bar graphs show FACS-based cell cycle distribution at 24, 48, and 72 hours post-transfection as the average of three independent experiments. Error bars represent standard deviation. When compared to cells expressing only EGFP, by 72 hours post-transfection, the fraction of M2-APC expressing cells in G2/M increased by 2.4-fold, and the S phase decreased by 77%; the fraction of M3-APC expressing cells in G2/M increased by 2-fold, and the S phase decreased by 31%. (C) Live cell scoring for mitotic indices of 100 EGFP-positive cells 24 hours and 48 hours post-transfection. Three independent experiments revealed no change in the mitotic population when cells expressed M2 (24 hours, 2±1, *p* = 0.74; 48 hours, 3±3, *p* = 0.19) or M3-APC (24 hrs, 6±3, *p* = 0.67; 48 hrs, 1±1, *p* = 0.29), as compared to cells expressing EGFP alone (24 hours, 3±3; 48 hours 4±2).

M2- and M3-APC each bind endogenous topo IIα in cells (Figure 1B) and purified topo IIα *in vitro* (Figure 2). Thus, we expressed M3-APC in HCT116βw cells (HCT116 cells that contain only a wild-type allele of β-catenin) to determine whether this expression altered cell cycle progression. Cell cycle distribution was determined by FACS analysis of living cells labeled with propidium iodide. Similar to what was previously seen using M2-APC [18], cells expressing M3-APC progressively accumulated in the G2/M phases of the cell cycle, while cells expressing EGFP did not (Figure 3A and B, Table S1). When compared to cells expressing EGFP alone, cells expressing M3-APC for 72 hours showed a 2-fold increase in G2/M distribution and a 31% decrease in S phase distribution; cells expressing M2-APC showed a 2.4-fold increase in G2/M distribution and a 77% decrease in S phase distribution. We conclude that expression of M2- or M3-APC leads to cell cycle accumulation in G2/M. Of note, the reduction in S phase cells seen upon expression of M2- or M3-APC, suggested a second cell cycle delay prior to S phase, likely in G1. This apparent delay in G1 is consistent with a previous observation that APC regulates the G1-S transition [13].

M2-APC expression elicits cell accumulation in the G2 phase rather than in mitosis [18]. FACS analysis does not distinguish between the G2 and M cell cycle populations. Thus, to determine whether expression of M3-APC also resulted in G2 accumulation, we determined the mitotic indices in living or fixed M3-APC-expressing cells at 24 and 48 hours post-transfection. We found no significant expansion of the mitotic population in M3-APC expressing cells by either counting phospho-histone H3 positive cells (data not shown) or by estimating the percentage of living cells displaying mitotic figures as visualized with Hoechst stain (Figure 3C and Table S2). Therefore, as previously reported for M2-APC, the expanded G2/M population of M3-APC-expressing cells determined by FACS analysis represents an accumulation in G2, not in M. Together, these results demonstrate that M3-APC and M2-APC each induce G2 cell cycle arrest when expressed in a colon epithelial cell line with full-length APC.

### G2 arrest triggered by M2- and M3-APC is not dependent on p53

**Figure 4:**
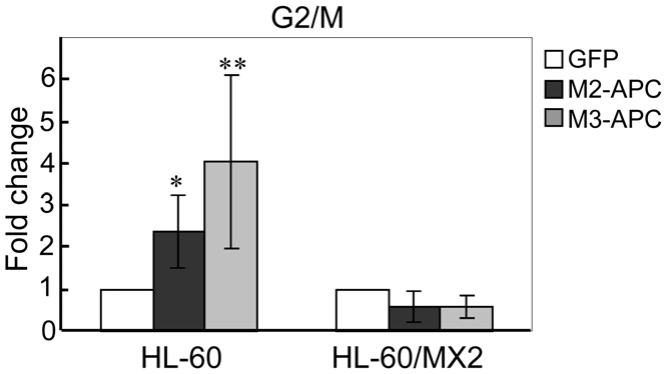
Expression of M2- or M3-APC results in G2/M accumulation in HL60 but not HL60/MX2 cells. Graph shows the G2/M population from HL-60 and HL-60/MX2 cells expressing EGFP-M2- or M3-APC for 48 hours. Parental HL60 cells exhibit a 2-fold increase in G2/M when expressing M2-APC and a 4-fold increase when expressing M3-APC. A student's t-test demonstrated that these increases in G2/M were significant for M2 (* p = 0.02) and M3 (** p = 0.03), compared to cells expressing only EGFP. HL60/MX2 cells exhibit a decrease in G2/M when expressing M2- or M3-APC. However, these differences were not significant: M2 (*p* = 0.06) and M3 (*p* = 0.48), compared to cells expressing only EGFP. For both cell lines, 10,000 EGFP-positive cells were analyzed from three independent experiments.

Tumor suppressor p53 participates in various pathways that regulate the G2/M transition (reviewed in [28]). To determine if p53 is required for the M2- and M3-APC-mediated G2 arrest, we determined the consequences of M2- or M3-APC expression in the promyelocytic leukemia cell line, HL-60. HL-60 cells express full-length APC protein (data not shown) but are null for p53 [29]. When either M2- or M3-APC was expressed in HL-60 cells, the G2/M population increased significantly (Figure 4A and Table S3). M2-APC expression resulted in a near doubling of the G2/M population, while M3-APC expression resulted in a tripling of the G2/M population. The G2 arrest in HL-60 cells indicates that the impact of M2- and M3-APC on the cell cycle is not restricted to colon epithelial cell lines. Moreover, the G2 cell cycle arrest triggered by expression of either M2- or M3-APC is not dependent on p53.

### Cells deficient in topo II do not arrest in G2 following expression of either M2- or M3-APC

Based on our data, we hypothesize that exogenous M2- and M3-APC each interact with endogenous topo IIα, resulting in a p53-independent cell cycle arrest in G2. Ideally, to demonstrate that this APC-mediated cell cycle regulation is dependent upon topo IIα, we would eliminate topo IIα from the analyzed cells. Unfortunately, topo IIα is an essential protein and its perturbation typically results in cell cycle delay followed by cell death [30]. Although no cultured mammalian cell line completely lacks topo IIα, there are cell line variants such as HL-60/MX2 cells with compromised topo IIα activity. HL-60/MX2 cells were originally generated by selecting for resistance to the topo II inhibitor mitoxantrone [31]. Thus, compared to parental HL-60 cells, they are 195-fold less sensitive to drugs that target topo II [32], [33], [34]. This resistance has been attributed to the observation that HL-60/MX2 cells express no topo IIβ, only a low level of topo IIα, and a truncated topo IIα with reduced activity and aberrant subcellular localization [32], [33], [34]. In contrast to the parental HL-60 cells, we observed that HL-60/MX2 cells expressing M2- or M3-APC showed no increase in the G2/M population (Figure 4 and Table S3). Rather, the slight decrease in G2/M and increase in the S population of M2- and M3-APC-expressing cells were not significantly different from the EGFP-expressing HL-60/MX2 cells. Taken together with our observation that M2- and M3-APC interact preferentially with topo IIα rather than topo IIβ (Figure 1B), we conclude that topo IIα is required for M2- or M3-APC-triggered cell cycle arrest in G2.

### Cells with truncated APC do not arrest in G2 following expression of M2- or M3-APC

**Figure 5:**
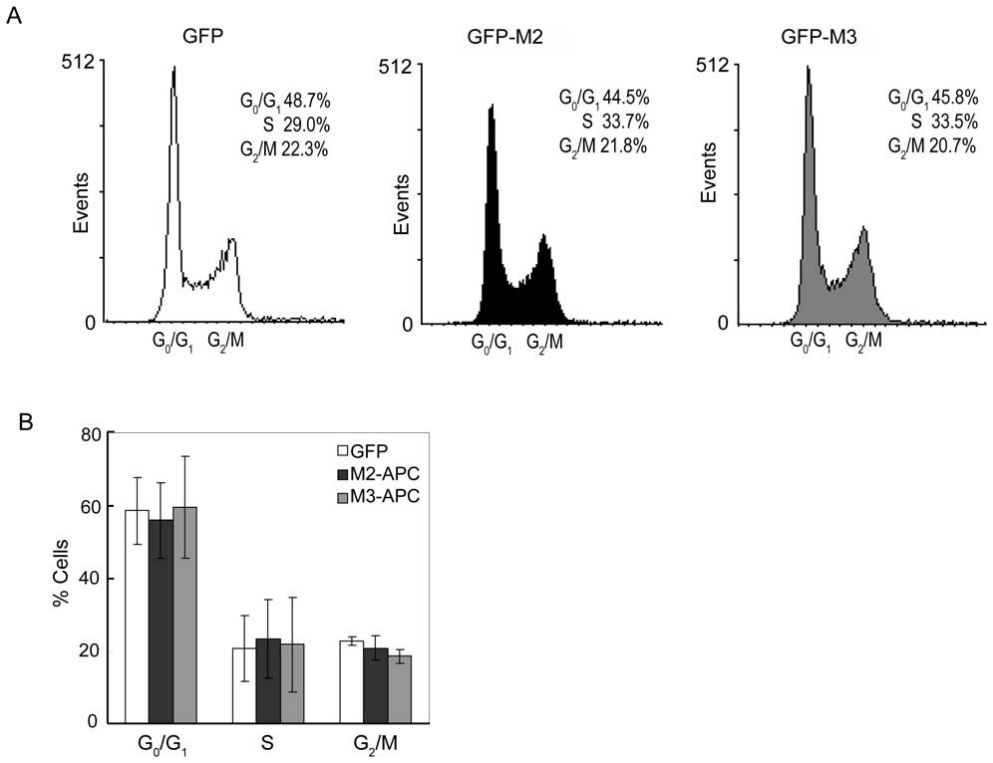
Expression of M2- or M3-APC does not affect cell cycle progression of colon cancer cells with endogenous truncated APC. (A) SW480 cells with endogenous truncated APC and expressing M2-, M3-APC or EGFP alone have similar cell cycle distributions. Histograms showing FACS analysis of EGFP-positive cells at 48 hours post-transfection. (B) Bar graphs show the average cell cycle distribution of SW480 cells expressing EGFP, M2 or M3-APC from three independent experiments. Error bars represent standard deviation.

The majority of somatic *APC* mutations in colon cancers result in overexpression of a truncated protein that includes all of the M2-APC region and part of M3-APC (Table 1). We examined the cell cycle profile of SW480 cells which express an endogenous APC protein truncated at amino acid 1368. In SW480 cells, expression of either M2- or M3-APC did not lead to G2 arrest, or any other alterations in the cell cycle phases (Figure 5 and Table S4).

**Table 1:** Correlation of karyotype with APC status and presence of M2 and M3-APC in some commonly used colorectal cancer cell lines.

Cell Line	APC mutation 1^a^	APC mutation 2^a^	Inclusion of M2/M3 in APC truncation	Karyotype	Reference
C106	1238	1490	M2	79	[47]
C70	1309	LOH	M2	115–130	[47], [48]
C84	1451	2843	M2	56	[47]
C99	1367	LOH	M2	52	[47]
CaCo/Caco2/TC7	1367	LOH	M2	96	[47]
CoLo205	1554	2843	M2	68–75	[47]
COLO320	810	LOH	None	45–58, 53	[47], [49]
DLD-1/HCT15	1417	LOH	M2	44–47	[47], [49], [50], [51]
GP2D	1444	LOH	M2	45–47	[47], [48], [50]
HT29	853	1555	M2	69–73	[47], [48], [49]
HT55	1131	1308	M2	80	[47]
LoVo	1114	1429	M2	47–57	[47], [48], [49], [50]
LS1034	1309	LOH	most of M2	77	[47]
LS411	789	1556	M2	70–76	[47], [48]
SKCO1	1317	1443	M2	70–80	[50]
SW1417	1450	LOH	M2	66–71	[47], [48]
SW403	1197	1278	most of M2	60–65; 68	[47], [48]
SW480	1368	LOH	M2	54–58	[47], [48], [49]
SW620	1338	N/D	M2	45–57	[47], [48], [50]
SW837	1450	LOH	M2	38–40	[47], [48]
SW948	1114	1429	M2	67	[47]
T84	1488	LOH	M2	47–57	[50]
VACO4A	1354	LOH	M2	60–65	[47], [48]
VACO5	1419	1554	M2	43–47	[47], [48]
HCA7	2843	2843	N/A	42–43	[47], [48]
HCT116	2843	2843	N/A	43–46	[47], [48], [49], [51]
LS174T	2843	2843	N/A	47,45, 46–47	[47], [48], [49]
RKO^b^	2843	2843	N/A	45–47	[49], [51]
SW48	2843	2843	N/A	46–47	[47], [49]
LS180	2843	2843	N/A	45	[47]

a APC status from [52].

b APC status from [53] LOH =  Loss of heterozygosity.

N/D = Not detected N/A  = Not applicable.

### G2 accumulation in M2- and M3-APC-expressing cells does not depend on β-catenin regulation

**Figure 6:**
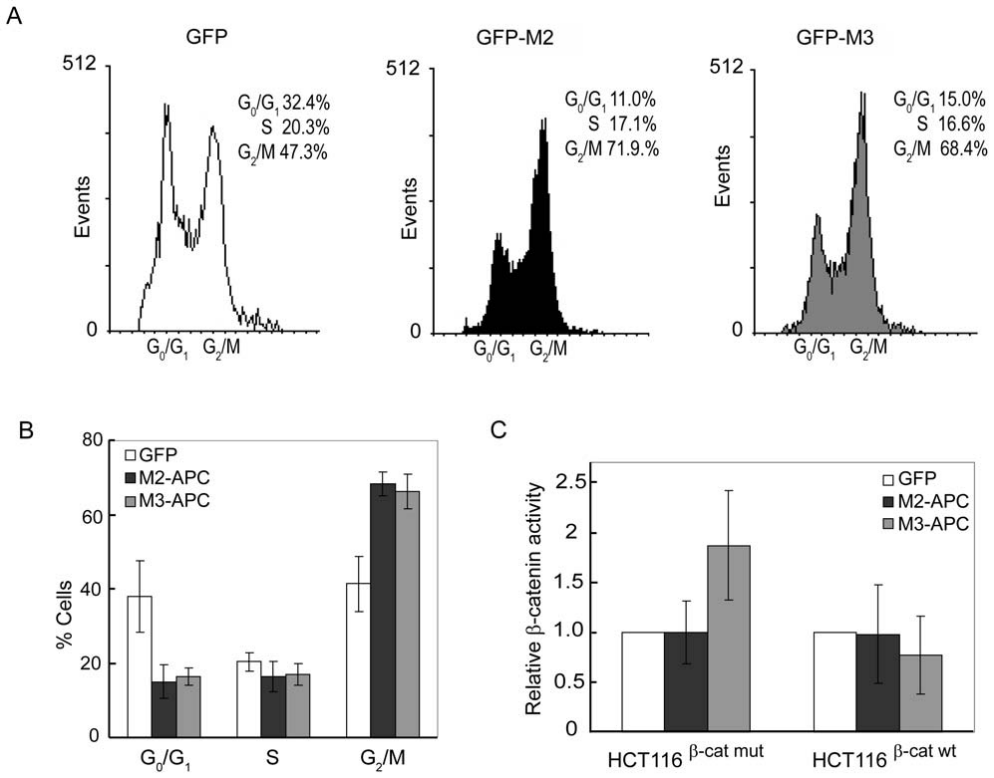
Mutant β-catenin does not compromise the ability of M2- or M3-APC to trigger G2 cell cycle arrest. (A) HCT116βm cells that express only stabilized β-catenin show accumulation in G2/M when expressing M2- or M3-APC. Histograms showing representative FACS displays of cell cycle distribution assessed by Hoechst blue staining at 48 hours post-transfection. Only EGFP-positive cells are displayed. (B) Bar graphs show FACS-based cell cycle distribution from three independent experiments. Error bars represent standard deviation. When compared to cells expressing only EGFP, by 48 hours post-transfection, the fraction of M2 or M3-APC expressing cells in G2/M increased by 1.7-and 1.6-fold, respectively. (C) Expression of M2-APC does not alter β-catenin activity in HCT116βw (β-cat wt) or HCT116βm (β-cat mut) cells; however, expression of M3-APC leads to distinct changes of β-catenin activity in the two cell lines. Luciferase activities were determined 48 hours post-transfection and activity of the β-catenin reporter construct (TOP-flash) was normalized against both pRL-TK Renilla activity and FOP-flash reporter activity. p values for HCT116βm (β-cat mut) cells are *p = 0.50* (M2) and *p = 0.03* (M3); and for HCT116βw (β-cat wt) cells are *p = 0.49* (M2) and *p = 0.39* (M3). Values are presented as average ± standard deviation for triplicate samples from three independent experiments.

A major function of the central region of tumor suppressor APC is to target, β-catenin for proteasome-mediated destruction. We have previously reported that expression of M2-APC does not alter β-catenin localization, level or activity and concluded that the G2 arrest triggered by M2-APC is not likely mediated by β-catenin [18]. Although both M2 and M3-APC bind β-catenin, only the M3-APC region is capable of targeting β-catenin for cytoplasmic destruction. To test if the G2 arrest triggered by M3-APC involves β-catenin regulation, we expressed M3-APC in HCT116βm cells which produce only stabilized β-catenin that can not be targeted for degradation [35]. HCT116βm cells expressing either M2 or M3-APC displayed a near doubling of the G2/M population with an accompanying decrease in the G0/G1 population when compared to HCT116βm cells expressing EGFP alone (Figure 6A and B and Table S5). Moreover, using a reporter construct to determine β-catenin activity as a transcription co-activator of LEF-1, it was demonstrated that expression of M3-APC led to increased β-catenin activity in HCT116βm cells, but slightly decreased β-catenin activity in HCT116βw cells (Figure 6*C*). This opposing effect of M3-APC on β-catenin activities in the two cell lines supports our conclusion that a decrease in β-catenin activity is not required for M3-APC-triggered G2/M cell cycle delay. Furthermore, expression of APC amino acids 1379–2080 in SW480 resulted in decreased β-catenin activity [36]. Thus, our finding that SW480 cells expressing M3-APC do not arrest in G2 (Figure 5) indicates that decreasing β-catenin activity is not sufficient to trigger a G2/M cell cycle delay.

### G2 arrest triggered by M2- and M3-APC is accompanied by increased aneuploidy

**Figure 7:**
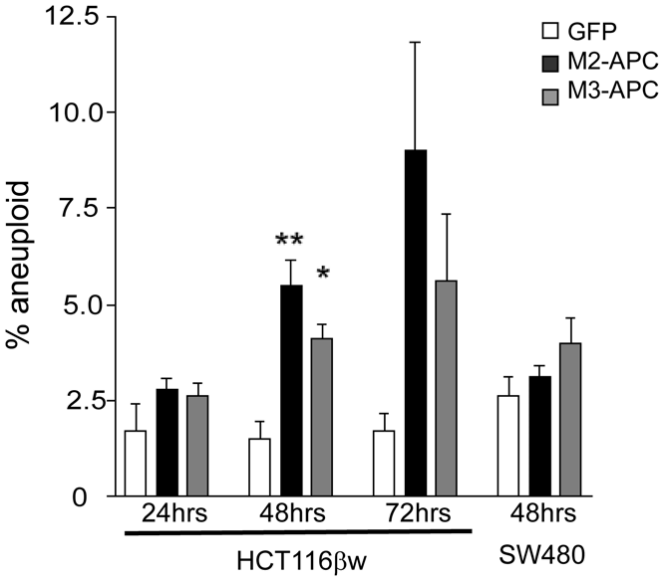
Expression of M2- or M3-APC results in increased aneuploidy in cells possessing full-length APC, but not in cells with truncated APC. Aneuploid cells were quantified by FACS analysis as shown in Figure 3 and Figure 5. The number of aneuploid HCT116βw cells steadily increased following expression of M2- or M3-APC. At 48 hours, there was a significant increase in aneuploidy (*p = 0.0070* for M2 and *p = 0.013* for M3) compared to cells expressing EGFP alone. There was not a significant increase in aneuploidy in SW480 cells transfected with M2- (*p = 0.16*) or M3-APC (*p = 0.09*).

Although derived from human colon cancer tissue, the HCT116 cell line retains a stable diploid karyotype. Not only did HCT116 cells expressing either M2- or M3-APC show progressive G2 arrest, but they displayed a significant increase in aneuploidy (Figure 7 and Table S1). In contrast, SW480 cells are originally aneuploid [Table 1]. Expression of either M2- or M3-APC in SW480 cells did not lead to a significant increase in aneuploidy (Figure 7 and Table S4).

## Discussion

In this study, we identified a novel topo IIα binding domain (M3) in the central region of APC that enhances both decatenation and relaxation activities of purified topo IIα. Cells expressing M2- or M3-APC accumulated in the G2 phase of the cell cycle and showed increased aneuploidy; however, this result was not observed in cells with endogenous truncated APC missing part of the M3 domain. The G2 arrest was also independent of p53 but was dependent on topo IIα. Our data indicate the central region of APC interacts with topo IIα and thereby regulates G2-M cell cycle progression.

### M2 and M3-APC each trigger G2 cell cycle arrest independent of topo IIβ

Topo IIα and topo IIβ are 75% identical in protein sequence and share some functional similarity in promoting DNA topology changes. However, successful generation of cell lines and mouse models completely lacking topo IIβ [24], [25], [26] indicates that topo IIβ is dispensable for cell cycle progression. In contrast, topo IIα is essential for cell cycle control. Our finding that M2- and M3-APC interact with endogenous topo IIα but not topo IIβ (Fig. 1B) is consistent with our previous observation that full-length endogenous APC interacts with endogenous topo IIα but not topo IIβ [18]. Based on this information, we conclude that APC-mediated G2 cell cycle arrest is not dependent on topo IIβ.

### Abnormal nuclear morphology is not associated with G2 arrest

We previously reported that expression of M2-APC in HCT116βw cells leads to abnormal nuclear morphology and G2 cell cycle arrest [18]. In contrast, although expression of M3-APC in HCT116βw cells also resulted in G2 arrest, the abnormal nuclear morphology was not observed until 72 hours post-transfection (data not shown). Since both M2 and M3-APC interact with and affect topo IIα, it seems most likely that the altered nuclear morphology observed shortly after M2-APC expression is topo IIα-independent. Furthermore, the abnormal nuclear morphology was also seen in SW480 cells [18] which do not undergo a G2 arrest following expression of either M2- or M3-APC (Figure 5). Thus, the abnormal nuclear morphology is not associated with G2 arrest.

### M2- and M3-APC are not identical in their interaction with topo IIα

Although both M2- and M3-APC bind topo IIα and trigger G2 cell cycle arrest when expressed in HCT116βw, HCT116βm, or HL60 cells, the cellular response to the two fragments is not identical. Altered nuclear morphology was observed 24 hours after expression of M2-APC [18]. In contrast, nuclear morphological alterations were not observed until 72 hours after expression of M3-APC (data not shown). In HCT116βw cells, expression of M2-APC for 72 hours resulted in a more robust G2 cell cycle accumulation than did expression of M3-APC (Figure 3B). In HL60 cells, expression of M3-APC resulted in a more robust G2 cell cycle accumulation than did expression of M2-APC (Figure 4). In transfected HCT116βw cells, M2-APC protein levels were approximately 3-fold higher than that of full-length endogenous APC and 2.7-fold higher than M3-APC levels (data not shown). However, M2- and M3-APC appeared able to co-precipitate equivalent amounts of endogenous topo IIα (Figure 1B). In general, it appeared that transfected HCT116βw cells expressed more M2-APC than M3-APC, but M3-APC was better able to bind endogenous topo IIα than M2-APC. Experiments using purified non-overlapping recombinant M2- and M3-APC fragments revealed that M3-APC enhanced the decatenation activity of purified topo IIα at a lower molar concentration than M2-APC (Fig. 2A). The opposite was observed in *in vitro* relaxation assays, where M2-APC appeared to enhance the ability of topo IIα to relax supercoiled DNA more effectively than M3-APC (Fig. 2C). Together, these observations are consistent with M2- and M3-APC binding to different regions of topo IIα and thus modifying topo IIα activity by slightly different mechanisms. Further investigation is required to identify the specific topo IIα binding sites and delineate the underlying mechanisms.

### Potential molecular mechanism and physiological relevance

Over 60% of FAP polyps display aneuploidy [2], [3], [4]. It has been proposed that APC mutations contribute to chromosomal instability (CIN) through loss of spindle-kinetochore attachment or misregulation of the cytoskeleton [for review, see [37]]. More recently, an association of truncated APC fragments with mitotic checkpoint protein Mad2 was reported to inactivate the mitotic checkpoint, thus providing another potential mechanism for CIN [38]. We propose a novel mechanism that might contribute to increased aneuploidy following mutation of APC. Our model is based on evidence that middle fragments of APC bind to topo IIα, affect topo IIα activity, and result in G2 cell cycle accumulation and increased aneuploidy when expressed exogenously.

We propose that expression of M2- or M3-APC causes altered topo IIα activity, thus activating the G2 decatenation checkpoint, which leads to G2 arrest. Aneuploid cells would result from altered topo IIα activity in the small percentage of mitotic cells that escape the G2 decatenation checkpoint. The G2 decatenation checkpoint is vital for cell cycle control and genomic integrity. Cells lacking the G2 decatenation checkpoint become aneuploid [39], [40]. A variety of topo II inhibitors have been shown to arrest cells in G2 by activating the G2 decatenation checkpoint [23]. It is possible that only cells with an intact G2 decatenation checkpoint can be arrested in G2 by expression of middle regions of APC. Consistent with this hypothesis, all cell lines we observed to undergo G2 arrest upon M2 or M3-APC expression possessed an intact decatenation checkpoint [41], [42]. HL60-MX2 cells have no decatenation checkpoint [32], [33], [34] and did not arrest in G2 (Fig. 4). In our study, the display of aneuploidy in cells expressing M2- or M3-APC increased steadily over time (Figure 7). We suggest that the basis for this aneuploid accumulation is compromised topo IIα activity in cells that escape the G2 decatenation checkpoint. We further predict that in FAP patients, truncated APC fragments which contain the M2 region would similarly interact with topo IIα and this might result in aneuploidy. In support of this prediction, a literature review of colorectal cancer cell lines reveals a general trend that cells with full-length APC are diploid with stable karyotypes (Table 1). In contrast, cell lines that express a truncated APC that includes M2-APC are mostly aneuploid, with very few exceptions. Our results provide a potential explanation for the presence of aneuploidy in early FAP adenomas. Further experiments comparing topo IIα activity in colon cancer cell lines harboring various truncating *APC* mutations are needed to establish a direct link between topo IIα activity and aneuploidy. The current study expands the repertoire of molecular factors implicated in the pathogenesis of colorectal cancer, illuminating new areas for future development of treatment strategies.

## Materials and Methods

### Cell culture and DNA constructs

HCT116βw (containing one wild-type allele of β-catenin) and HCT116βm (containing one mutant allele of β-catenin) cells (a gift from Dr. Bert Vogelstein) and SW480 (ATCC) were grown in McCoy's 5A medium (Gibco) supplemented with 10% FBS (Hyclone). HL60 cells (ATCC) were grown in Iscove's Modified Dulbecco's Medium (ATCC) supplemented with 20% FBS (Hyclone). HL60/MX2 (ATCC) were grown in RPMI 1640 medium (Cellgro) supplemented with 10% FBS. Expression constructs for APC fragments fused to EGFP were kindly provided by Dr. Naoki Watanabe and have been described previously [43]. His and S dual-tag fused M2-APC was made as described [18]. To generate recombinant N-terminal His and S dual-tag fused APC fragment M3, the corresponding cDNA for APC (amino acid 1330–2058) was amplified using PCR and subcloned into a pET-30a(+) vector.

### Immunoprecipitation and immunoblots

HCT116βw cells were transfected using Lipofectamine2000 reagent according to the manufacturer's protocol (Invitrogen). Transfection efficiencies estimated by FACs analysis were, on average, 48% for EGFP-M2-APC and 62% for EGFP-M3-APC. Estimated relative levels of M2-APC, M3-APC and full-length endogenous APC in whole cell lysates were 1.6∶0.7∶1. Immunoprecipitation (IP) and immunoblots (IB) were performed using anti-GFP pAb (Invitrogen) as described [18]. Immunoblots were probed with the following antibodies: anti-β-catenin (1∶2000, Sigma); anti-topo IIα (1∶1000, Research Diagnostics, Inc.); anti-topo IIβ (1∶1000, Santa Cruz); anti-GFP pAb (1∶1000, Invitrogen); and anti-α-tubulin (1∶2000, Oncogene).

### Electroporation and FACS analysis

Cells grown on plastic were treated with trypsin to obtain a single cell suspension. A total of 2 µg of EGFP, EGFP fused M2-, or M3-APC expression plasmid were electroporated using Nucleofector I (Amaxa) according to the manufacturer's protocol. Electroporation programs used were: HCT116βw (program D-32), HCT116βm (program D-32), HL60 (program T-19), and HL60/MX2 (program X-03). SW480 cells were transfected with Metafectine (Scientifix, Australia). Forty-eight hours post-transfection, single cells in suspension were stained with 0.5 µg/ml Hoechst 33342 (Invitrogen) for 30 minutes at 37°C. FACS analysis was performed using both UV and 488 nm lasers on a 5-laser BD LSRII flow cytometry (BD Bioscience). Ten thousand EGFP-positive cells were collected for each sample. Data were analyzed using BD FACSDiva Software (BD Bioscience) and plotted using WinMDI 2.9.

### Recombinant proteins and topo IIα relaxation and decatenation assays

Recombinant S-tag fused M2-APC (amino acid 1000–1326) and M3-APC (amino acid 1330–2058) were generated as described [18]. BSA (Sigma) was diluted in S-tagged APC protein dilution buffer (20 mM Hepes pH 7.8, 100 mM NaCl). Recombinant human topo IIα and topo IIβ were made as described [44], [45]. *In vitro* topo IIα relaxation and decatenation assays were performed as described [20].

### Antibodies and immunofluorescence

Cells transfected with EGFP or EGFP-fused M3-APC were fixed with 4% paraformaldehyde, and immunostaining was performed using anti-phospho-histone H3 (1∶500, Upstate) as described [46]. One hundred EGFP-positive cells were randomly chosen, and only cells also positive for phospho-histone-H3 were counted. As a second method to determine mitotic indices, living cells were stained with 0.5 µg/ml Hoechst 33342 at 24 and 48 hours post-transfection and mitotic figures were counted for 100 cells in each category. The mitotic indices are presented as an average ± s.d. of three independent experiments.

### Reporter gene assay

HCT116βw and HCT116βm cells grown in 24-well plates were co-transfected using Metafectine reagent (Scientifix, Australia) with 2 µg of the EGFP-M2-APC, EGFP-M3-APC or EGFP expression construct, 100 ng of the TCF-reporter construct SuperTOP-flash or FOPflash (Upstate Biotechnology, Lake Placid, NY), and 50 ng of the pRL-TK Renilla luciferase construct (Promega, WI) as a control to normalize for transfection efficiency. After 48 hours, cells were harvested and luciferase activities were determined using the Dual-Luciferase® assay system (Promega) and a Turner Designs TD-20/20 luminometer. SuperTOP-flash and FOPflash luciferase activities were first normalized by pRL-TK Renilla luciferase, and then the normalized SuperTOP-flash luciferase activity was divided by normalized FOPflash luciferase activity to calculate relative β-catenin activity.

## Supporting Information

Table S1 Cell cycle distribution in HCT116 βw cells expressing GFP, M2-APC, or M3-APC. Transfected cells were stained with Hoechst blue, and the cell cycle distribution G0/G1 (2N), S (between 2N and 4N), and G2/M (4N) was determined by FACS at three time points post-transfection. For each transfection, 10,000 GFP-positive cells were analyzed. Table shows the average from three independent experiments.
(0.04 MB DOC)

Table S2 Mitotic indices of HCT116βw cells expressing GFP, M2-APC, or M3-APC. Live GFP, M2-APC, and M3-APC expressing cells at 48 hours post-transfection were stained with Hochest blue. Mitotic cells were counted according to DNA morphology from 100 randomly selected GFP positive cells. Table shows the average from three independent experiments. p values were calculated by comparing M2 or M3-APC expressing cells to GFP expressing cells using student t test.
(0.03 MB DOC)

Table S3 Cell cycle distribution of parental HL60 and HL60/MX2 cells expressing GFP, M2-APC, or M3-APC. Cell cycle distributions of GFP, M2-APC, and M3-APC expressing cells at 48 hours post-transfection. For each transfection, 10,000 GFP-positive cells were analyzed. Table shows the average from three independent experiments.
(0.03 MB DOC)

Table S4 Cell cycle distribution of SW480 cells expressing GFP, M2-APC, or M3-APC. Cell cycle distributions of GFP, M2-APC, and M3-APC expressing cells at 48 hours post-transfection. For each transfection, 10,000 GFP-positive cells were analyzed. Table shows the average from three independent experiments. For aneupoid cells, p values for M2-APC is 0.16, and for M3-APC is 0.09.
(0.03 MB DOC)

Table S5 Cell cycle distribution of HCT116βm cells expressing GFP, M2-APC, or M3-APC. Cell cycle distributions of GFP, M2-APC, and M3-APC expressing cells at 48 hours post-transfection. For each transfection, 10,000 GFP-positive cells were analyzed. Table shows the average from three independent experiments.
(0.03 MB DOC)
