# Supplementary material for: Topoisomerase IIα Binding Domains of Adenomatous Polyposis Coli Influence Cell Cycle Progression and Aneuploidy
Source: PLoS One. 2010 Apr 2;5(4):e9994. doi: 10.1371/journal.pone.0009994 (PMC2848841; doi:10.1371/journal.pone.0009994)
Supplement: Table S1 — Cell cycle distribution in HCT116 βw cells expressing GFP, M2-APC, or M3-APC. Transfected cells were stained with Hoechst blue, and the cell cycle distribution G0/G1 (2N), S (between 2N and 4N), and G2/M (4N) was determined by FACS at three time points post-transfection. For each transfection, 10,000 GFP-positive cells were analyzed. Table shows the average from three independent experiments. (0.04 MB DOC) [file pone.0009994.s001.doc]

**Table S1. Cell cycle distribution in HCT116βw cells expressing GFP, M2-APC, or M3-APC**

| **HCT116βw** | 24 h | G0/G1 (%) | S (%) | G2/M (%) | Aneuploid |
| --- | --- | --- | --- | --- | --- |
| 24 hours | GFP | 44.3 +/- 4.7 | 33.4 +/- 1.3 | 22.3 +/- 4.8 | 1.7 +/- 1.21 |
|  | M2-GFP | 44.1 +/- 1.7 | 27.9 +/- 1.6 | 28.0 +/- 2.8 | 2.8 +/- 0.5 |
|  | M3-GFP | 41.5 +/- 4.6 | 31.0 +/- 1.6 | 27.5 +/- 3.1 | 2.6 +/- 0.6 |
| 48 hours | GFP | 43.8 +/- 2.8 | 34.5 +/- 4.3 | 21.7 +/- 3.3 | 1.5 +/- 0.8 |
|  | M2-GFP | 43.3 +/- 5.4 | 20.6 +/- 0.6 | 36.1 +/- 5.9 | 5.5 +/- 1.1 |
|  | M3-GFP | 43.9 +/- 3.5 | 23.2 +/- 2.9 | 32.9 +/- 1.6 | 4.1 +/- 0.7 |
| 72 hours | GFP | 41.1 +/- 2.8 | 39.7 +/- 3.4 | 19.2 +/- 3.4 | 1.7 +/- 0.8 |
|  | M2-GFP | 43.1 +/- 6.9 | 9.2 +/- 6.6 | 47.7 +/- 2.5 | 9.0 +/- 4.9 |
|  | M3-GFP | 33.6 +/- 9.5 | 27.2 +/- 6.6 | 39.2 +/- 3.0 | 5.6 +/- 3.0 |
