# Supplementary material for: Topoisomerase IIα Binding Domains of Adenomatous Polyposis Coli Influence Cell Cycle Progression and Aneuploidy
Source: PLoS One. 2010 Apr 2;5(4):e9994. doi: 10.1371/journal.pone.0009994 (PMC2848841; doi:10.1371/journal.pone.0009994)
Supplement: Table S2 — Mitotic indices of HCT116βw cells expressing GFP, M2-APC, or M3-APC. Live GFP, M2-APC, and M3-APC expressing cells at 48 hours post-transfection were stained with Hochest blue. Mitotic cells were counted according to DNA morphology from 100 randomly selected GFP positive cells. Table shows the average from three independent experiments. p values were calculated by comparing M2 or M3-APC expressing cells to GFP expressing cells using student t test. (0.03 MB DOC) [file pone.0009994.s002.doc]

**Table S2. Mitotic indices of HCT116βw cells expressing GFP, M2-APC, or M3-APC**

|  |  | Mitotic± s.d. (%) | *p* value |
| --- | --- | --- | --- |
| 24 hours | GFP | 3 ± 3 | N/A |
|  | M2-GFP | 2 ± 1 | 0.74 |
|  | M3-GFP | 6 ± 3 | 0.19 |
| 48 hours | GFP | 4 ± 2 | N/A |
|  | M2-GFP | 3 ± 3 | 0.67 |
|  | M3-GFP | 1 ± 1 | 0.29 |
