# Supplementary material for: Topoisomerase IIα Binding Domains of Adenomatous Polyposis Coli Influence Cell Cycle Progression and Aneuploidy
Source: PLoS One. 2010 Apr 2;5(4):e9994. doi: 10.1371/journal.pone.0009994 (PMC2848841; doi:10.1371/journal.pone.0009994)
Supplement: Table S3 — Cell cycle distribution of parental HL60 and HL60/MX2 cells expressing GFP, M2-APC, or M3-APC. Cell cycle distributions of GFP, M2-APC, and M3-APC expressing cells at 48 hours post-transfection. For each transfection, 10,000 GFP-positive cells were analyzed. Table shows the average from three independent experiments. (0.03 MB DOC) [file pone.0009994.s003.doc]

**Table S3. Cell cycle distribution of parental HL60 and HL60/MX2 cells expressing GFP, M2-APC, or M3-APC**

| **HL60** | G0/G1 (%) | S (%) | G2/M (%) |
| --- | --- | --- | --- |
| GFP | 81.8 ± 5.4 | 13.0 ± 3.8 | 5.2 ± 2.0 |
| M2-GFP | 82.2 ± 7.8 | 7.8 ± 6.8 | 10 ± 2.1 |
| M3-GFP | 67.3 ± 4.1 | 14.8 ± 5.1 | 17.9 ± 3.3 |

| **HL60/MX2** | G0/G1 (%) | S (%) | G2/M (%) |
| --- | --- | --- | --- |
| GFP | 65.7 ± 1.0 | 21.7 ± 1.8 | 12.6 ± 1.0 |
| M2-GFP | 53.3 ± 15.9 | 39.4 ± 14.2 | 7.3 ± 4.0 |
| M3-GFP | 50.5 ± 9.7 | 42.3 ± 10.1 | 7.2 ± 2.8 |
