# Supplementary material for: Topoisomerase IIα Binding Domains of Adenomatous Polyposis Coli Influence Cell Cycle Progression and Aneuploidy
Source: PLoS One. 2010 Apr 2;5(4):e9994. doi: 10.1371/journal.pone.0009994 (PMC2848841; doi:10.1371/journal.pone.0009994)
Supplement: Table S4 — Cell cycle distribution of SW480 cells expressing GFP, M2-APC, or M3-APC. Cell cycle distributions of GFP, M2-APC, and M3-APC expressing cells at 48 hours post-transfection. For each transfection, 10,000 GFP-positive cells were analyzed. Table shows the average from three independent experiments. For aneupoid cells, p values for M2-APC is 0.16, and for M3-APC is 0.09. (0.03 MB DOC) [file pone.0009994.s004.doc]

**Table S4. Cell cycle distribution of SW480 cells expressing GFP, M2-APC, or M3-APC**

| **SW480** | G0/G1 (%) | S (%) | G2/M (%) | Aneuploid (%) |
| --- | --- | --- | --- | --- |
| GFP | 58.6 ± 9.2 | 20.7 ± 9.0 | 22.7 ± 1.1 | 2.6 ± 0.9 |
| M2-GFP | 55.9± 10.3 | 23.3 ± 10.8 | 20.8 ± 3.4 | 3.1 ± 0.5 |
| M3-GFP | 59.5 ± 14.0 | 21.8± 12.9 | 18.6 ± 1.9 | 4.0 ± 1.1 |
