# Supplementary material for: Topoisomerase IIα Binding Domains of Adenomatous Polyposis Coli Influence Cell Cycle Progression and Aneuploidy
Source: PLoS One. 2010 Apr 2;5(4):e9994. doi: 10.1371/journal.pone.0009994 (PMC2848841; doi:10.1371/journal.pone.0009994)
Supplement: Table S5 — Cell cycle distribution of HCT116βm cells expressing GFP, M2-APC, or M3-APC. Cell cycle distributions of GFP, M2-APC, and M3-APC expressing cells at 48 hours post-transfection. For each transfection, 10,000 GFP-positive cells were analyzed. Table shows the average from three independent experiments. (0.03 MB DOC) [file pone.0009994.s005.doc]

**Table S5. Cell cycle distribution of HCT116βm cells expressing GFP, M2-APC, or M3-APC**

| **HCT116βm** | G0/G1 (%) | S (%) | G2/M (%) |
| --- | --- | --- | --- |
| GFP | 38.0 ± 9.5 | 20.3 ± 2.5 | 41.3 ± 7.4 |
| M2-GFP | 15.0± 4.6 | 16.3 ± 4.0 | 68.3 ± 3.2 |
| M3-GFP | 16.3 ± 2.3 | 17.0± 3.0 | 66.3 ± 4.7 |
